# Connecting the use of innovative treatments and glucocorticoids with the multidisciplinary evaluation through rule-based natural-language processing: a real-world study on patients with rheumatoid arthritis, psoriatic arthritis, and psoriasis

**DOI:** 10.3389/fmed.2023.1179240

**Published:** 2023-06-14

**Authors:** Francesca Motta, Pierandrea Morandini, Fiore Maffia, Matteo Vecellio, Antonio Tonutti, Maria De Santis, Antonio Costanzo, Francesca Puggioni, Victor Savevski, Carlo Selmi

**Affiliations:** ^1^Department of Biomedical Sciences, Humanitas University, Pieve Emanuele, Milan, Italy; ^2^Division of Rheumatology and Clinical Immunology, IRCCS Humanitas Research Hospital, Rozzano, Milan, Italy; ^3^Artificial Intelligence Center, IRCCS Humanitas Research Hospital, Rozzano, Milan, Italy; ^4^Wellcome Centre for Human Genetics, University of Oxford, Oxford, United Kingdom; ^5^Centro Ricerche Fondazione Italiana Ricerca Sull'Artrite (FIRA), Fondazione Pisana per la Scienza ONLUS, San Giuliano Terme (Pisa), Milan, Italy; ^6^Division of Dermatology, IRCCS Humanitas Research Hospital, Rozzano, Milan, Italy; ^7^Division of Asthma, Allergy and Personalized Medicine, IRCCS Humanitas Research Hospital, Rozzano, Milan, Italy

**Keywords:** artificial intelligence, rheumatology, inflammatory arthritis, electronic medical record (EMR), psoriatic arthritis

## Abstract

**Background:**

The impact of a multidisciplinary management of rheumatoid arthritis (RA), psoriatic arthritis (PsA), and psoriasis on systemic glucocorticoids or innovative treatments remains unknown. Rule-based natural language processing and text extraction help to manage large datasets of unstructured information and provide insights into the profile of treatment choices.

**Methods:**

We obtained structured information from text data of outpatient visits between 2017 and 2022 using regular expressions (RegEx) to define elastic search patterns and to consider only affirmative citation of diseases or prescribed therapy by detecting negations. Care processes were described by binary flags which express the presence of RA, PsA and psoriasis and the prescription of glucocorticoids and biologics or small molecules in each cases. Logistic regression analyses were used to train the classifier to predict outcomes using the number of visits and the other specialist visits as the main variables.

**Results:**

We identified 1743 patients with RA, 1359 with PsA and 2,287 with psoriasis, accounting for 5,677, 4,468 and 7,770 outpatient visits, respectively. Among these, 25% of RA, 32% of PsA and 25% of psoriasis cases received biologics or small molecules, while 49% of RA, 28% of PsA, and 40% of psoriasis cases received glucocorticoids. Patients evaluated also by other specialists were treated more frequently with glucocorticoids (70% vs. 49% for RA, 60% vs. 28% for PsA, 51% vs. 40% for psoriasis; *p* < 0.001) as well as with biologics/small molecules (49% vs. 25% for RA, 64% vs. 32% in PsA; 51% vs. 25% for psoriasis; *p* < 0.001) compared to cases seen only by the main specialist.

**Conclusion:**

Patients with RA, PsA, or psoriasis undergoing multiple evaluations are more likely to receive innovative treatments or glucocorticoids, possibly reflecting more complex cases.

## Introduction

To manage rheumatoid arthritis (RA) a multidisciplinary approach is encouraged given the systemic nature of the condition and the common coexistence of other immune-mediated diseases (1, 2). Similarly, psoriatic arthritis (PsA) and skin or nail psoriasis (3) require more than one specialist besides the rheumatologist and dermatologist and (4), with this approach being supported by current recommendations (5–10) and providing favorable effects on symptoms and functional consequences (11). Multidisciplinary care may ultimately impact on treatment choices, in particular the use of systemic and topic glucocorticoids and more innovative drugs such as biologics and targeted synthetic small molecules, thus reflecting a more complex case mix.

Artificial intelligence (AI), particularly natural language processing (NLP) and machine learning, allows to perform complex analysis on large datasets and to extract punctual information from unstructured data (12, 13). In clinical research these tools manifest high-performance computing ability to select, organize and analyze large amounts of data through electronic medical records to ultimately support and improve clinical practice, as shown in rheumatology (14–17). As a proof, machine learning has been used to identify multimorbidity patterns in RA, (18) and to estimate the cardiovascular risk in PsA (19).

Moving from the 5-year experience of the Humanitas ImmunoCenter in which different specialists contribute to the management of patients with chronic immune-mediated diseases, we applied data extraction tools to assess the impact of a multidisciplinary approach on treatment choices in RA, PsA, and psoriasis.

## Materials and methods

### Clinical setting

The ImmunoCenter at Humanitas was established in 2017 to provide high-quality care to patients with autoimmune and chronic inflammatory diseases, particularly inflammatory arthritis, bowel disease, and skin conditions, as well as allergy and asthma. The Center is based on a core of specialists dedicated to rheumatology, dermatology, gastroenterology focusing on inflammatory bowel disease (IBD), and pulmonology/allergy.

### Data selection

Clinical notes from hospital visits of patients with a terminated or ongoing care process within the Humanitas ImmunoCenter between 2017 and 2022 were included in the analysis. Data regarding the primary diagnosis, the presence of comorbidities, the associated conditions, and the treatments were extracted from the text of the electronic medical records. Innovative treatments of interest included anti-TNF-alpha (adalimumab, etanercept, golimumab, certolizumab pegol, infliximab), anti-IL-6 receptor (tocilizumab, sarilumab), rituximab, abatacept, anti-IL-23 (ustekinumab, guselkumab), anti-IL-17 (secukinumab, ixekizumab), anti-JAK (tofacitinib, baricitinib, upadacitinib, filgotinib), and apremilast; for each mechanism of action, all approved patented names were also used. In the case of glucocorticoids, both systemic and topic molecule names were included in the search. Text data underwent minimal pre-processing by the conversion of non-ASCII characters (e.g., HTML tags, accents, etc) to their ASCII version. Markers were extracted regarding the presence of diseases or drugs in the appropriate paragraphs using a rule-based approach (20–22), specifically by means of Regular Expressions (RegEx) (23). A regular expression is a sequence of characters that allows to define an elastic search pattern able to account for mistyping errors. RegEx were also used to detect negations. To compute performance indexes on RegEx, 200 sentences for each disease were manually annotated, divided into two groups of 100. One group contained sentences in which regex detected the presence of the disease, while the other group contained sentences in which regex detected a negation of the same disease. The computed metrics are precision, recall, F1 score and accuracy. After marker extraction, the analysis focused on those hospital encounters who reported a diagnosis of RA, PsA or psoriasis.

### Analytical tools

Care processes were described by binary flags expressing the presence of RA, psoriasis, or PsA, the prescription of glucocorticoids, and the available innovative therapies for each patient. To assess the association between features and categorical variables, univariate analyses were performed using chi-square tests when using categorical features and ranksum Mann–Whitney test when using continuous features. Mann–Whitney test was the preferred option to account for non-Gaussian distributions. A multivariate analysis was performed to evaluate the influence of the patient journey (in terms of number and type of visits) combined with confounding factors, such as gender and age, to predict prescriptions. For each disease, the analysis is repeated on different cohorts of patients: all without distinction, those followed only by the leading specialist, and those followed also by other specialists. This is to evaluate how differently these features relate to the prescription of glucocorticosteroids and small biologic molecules during the care process. The results were interpreted by means of the odds ratio obtained by the coefficients of a logistic regression fitted on such variables. To further study the association between variables, we trained a logistic regression to predict the outcomes related to treatments. The used main predictors are the number of visits and a binary flag stating if a patient has been visited by more specialists. The performance of this classification was evaluated with a 10-fold cross-validation stratifying the folds by the outcome.

All statistical analyses were performed using Python and *p* values <0.05 were considered as statistically significant.

## Results

### Dataset

Our natural language processing approach identified 1743 patients with RA (77% women, mean ± standard deviation age 59 ± 16 years), 1,359 patients with PsA (48% women, age 56 ± 14 years), and 2,287 patients with psoriasis (43% women, age 52 ± 16 years; Table 1). The marker extraction phase was evaluated via manual annotation: a total of 600 sentences were selected to verify the RegEx outcome. High performance allows us to say that marker extraction phase occurred with a good degree of reliability with a calculated accuracy exceeding 95%.

**Table 1:** Demographic and clinical features and use of glucocorticoids and biologics according to the number of visits in patients with RA, PsA, and psoriasis.

	Rheumatoid arthritis (*n* = 1,743)	Psoriatic arthritis (*n* = 1,359)	Psoriasis (*n* = 2,287)
Age (mean + SD)	58.6 + 15.8	55.9 + 14.1	51.6 + 15.7
Women (%)	76.9%	48.4%	42.8%
Total visits	5,677	4,468	7,770
Patients seen only by the leading specialist (*n*)	1,661 (95%)	1,206 (89%)	1964 (86%)
Visits of patients seen only by the leading specialist (*n*)	5,126	3,305	5,583
Visits per patient (median, IQR)	2 (1–4)	2 (1-4)*n*	1 (1–3)
Patients with any number of visits with the leading specialist			
Biologic prescriptions (*n*)	417 (25%)	386 (32%)	494 (25%)
Glucocorticoid prescriptions (*n*)	813 (49%)	338 (28%)	788 (40%)
Patients with < 3 visits with the leading specialist			
Biologics prescriptions (*n*)	9%	14%	5%
Glucocorticoid prescriptions (*n*)	41%	23%	36%
Patients with ≥ 3 visits with the leading specialist			
Biologics prescriptions (*n*)	51%	66%	67%
Glucocorticoid prescriptions (*n*)	63%	44%	51%
Patients seen by other specialists (*n*)	82 (5%)	153 (11%)	323 (14%)
Visits of patients seen by other specialists (*n*)	551	1,163	2,187
Visits per patient (median, IQR)	5 [3–9]	7 [3–10]	6 [3–9]
Biologic prescriptions to patients seen by other specialists	40 (49%)	98 (64%)	163 (51%)
Glucucorticoid prescriptions to patients seen by other specialists	57 (70%)	91 (60%)	165 (51%)

Continuous variables are expressed as mean + standard deviation or median (interquartile range).

### Analysis

Between 2017 and 2022, there were 5,677 visits for RA, 4468 for PsA, and 7,770 for psoriasis among different specialists. Most patients were seen only by the leading specialist (rheumatologist for RA and PsA, dermatologist for psoriasis), with 5% RA, 11% PsA and 14% psoriasis cases evaluated also by other core specialists, i.e., rheumatologists, dermatologists, gastroenterologists, allergists or pulmonologists (Table 1).

Among identified patients, 457 (26%) RA, 484 (35%) PsA, and 657 (29%) psoriasis cases were prescribed biologics or targeted synthetic molecules, while systemic or topic glucocorticoids were prescribed at least once to 870 (50%) RA, 429 (31%) PsA, and 953 (42%) psoriasis cases, in the vast majorty of cases by the leading specialist and only in the case of psoriasis including the beta-methasone topical formulations for over 90% of prescriptions (*data not shown*). Patients with RA, PsA, or psoriasis evaluated by more than one specialist were treated more frequently with glucocorticoids (70% vs. 49% for RA, *p* < 0.001; 60% vs. 28% for PsA, p < 0.001, 51% vs. 40% for psoriasis, *p* < 0.001) as well as with innovative drugs (49% vs. 25% for RA, *p* < 0.001; 64% vs. 32% in PsA, *p* < 0.001, 51% vs. 25% for psoriasis, *p* < 0.001; Table 1).

The multivariate analysis demonstrated that patients seen three or more times by any specialist received significantly more glucocorticoids prescriptions (OR 1.17 95% CI 1.12–1.21 for RA; OR 1.1 95% CI 1.07–1.14 for PsA; OR 1.06 95% CI 1.04–1.09 for psoriasis) and biologics or targeted synthetic treatments (OR 1.6 95% CI 1.52–1.68 for RA; OR 1.78 95% CI 1.67–1.91 for PsA; OR 1.86 95% CI 1.76–1.97 for psoriasis), while the age did not influence the prescription profiles (Table 2). Also, patients evaluated three or more times by the leading specialist received significantly more glucocorticoids prescriptions (OR 1.18 95% CI 1.13–1.23 for RA; OR 1.93, 95% CI 1.78–2.09 for PsA; OR 1.05 95% CI 1.02–1.08 for psoriasis) and biologics or targeted synthetic oral molecules (OR 1.62 95% CI 1.53–1.71 for RA; OR 1.93 95% CI 1.78–2.09 for PsA; OR 2.08 95% CI 1.93–2.23 for psoriasis), once again the prescription profile not being influenced by age (Table 2).

**Table 2:** Multivariate analysis of determinants of the prescription of glucocorticoids (left panels) or biologics and small molecules (right panels).

		Feature	Glucocorticoids	Biologics / small molecules
All specialists	Rheumatoid artritis	Number of visits	1.17 [1.12–1.21]	1.6 [1.52–1.68]
		Age	1.0 [1.0–1.0]	0.96 [0.96–0.97]
		Gender	0.5 [0.41–0.62]	0.63 [0.48–0.82]
	Psoriasic artritis	Number of visits	0.1 [1.07–1.14]	1.78 [1.67–1.91]
		Age	0.99 [0.98–0.99]	0.96 [0.95–0.96]
		Gender	0.57 [0.46–0.71]	0.76 [0.58–0.92]
	Psoriasis	Number of visits	1.06 [1.04–1.09]	1.86 [1.76–1.97]
		Age	0.99 [0.99–0.99]	0.95 [0.94–0.95]
		Gender	0.82 [0.7–0.97]	0.57 [0.45–0.72]
Leading specialist (dermatologist, rheumatologist, IBD-specialist, allergist)	Rheumatoid artritis	Number of visits	1.18 [1.13–1.23]	1.62 [1.53–1.71]
		Age	1.0 [1.0–1.0]	0.96 [0.96–0.97]
		Gender	0.5 [0.4–0.62]	0.59 [0.45–0.77]
	Psoriasic artritis	Number of visits	1.07 [1.02–1.12]	1.93 [1.78–2.09]
		Age	0.99 [0.98–0.99]	0.96 [0.96–0.96]
		Gender	0.5 [0.39–0.63]	0.77 [0.58–1.03]
	Psoriasis	Number of visits	1.05 [1.02–1.08]	2.08 [1.93–2.23]
		Age	0.99 [0.99–0.99]	0.94 [0.94–0.95]
		Gender	0.81 [0.68–0.97]	0.52 [0.4–0.68]
Other specialists	Rheumatoid Artritis	Number of visits	0.99 [0.89–1.1]	1.23 [1.5–1.82]
		Age	1.01 [1.03–1.05]	0.95 [0.93–0.98]
		Gender	0.44 [0.14–1.36]	1.62 [0.53–5.0]
	Psoriasic Artritis	Number of visits	1.04 [0.97–1.11]	1.45 [1.27–1.66]
		Age	0.99 [1.0–1.01]	0.97 [0.96–0.99]
		Gender	1.3 [0.69–2.44]	0.67 [0.31–1.45]
	Psoriasis	Number of visits	1.07 [1.01–1.12]	1.54 [1.4–1.7]
		Age	1.0 [0.99–1.0]	0.95 [0.94–0.96]
		Gender	0.84 [0.55–1.28]	0.91 [0.54–1.55]

For ≥3 visits, age (per incremental year) and female gender data are expressed as odds ratios and 95% confidence intervals for RA (upper row), PsA (middle row), and psoriasis (lower row).

**Figure 1:**
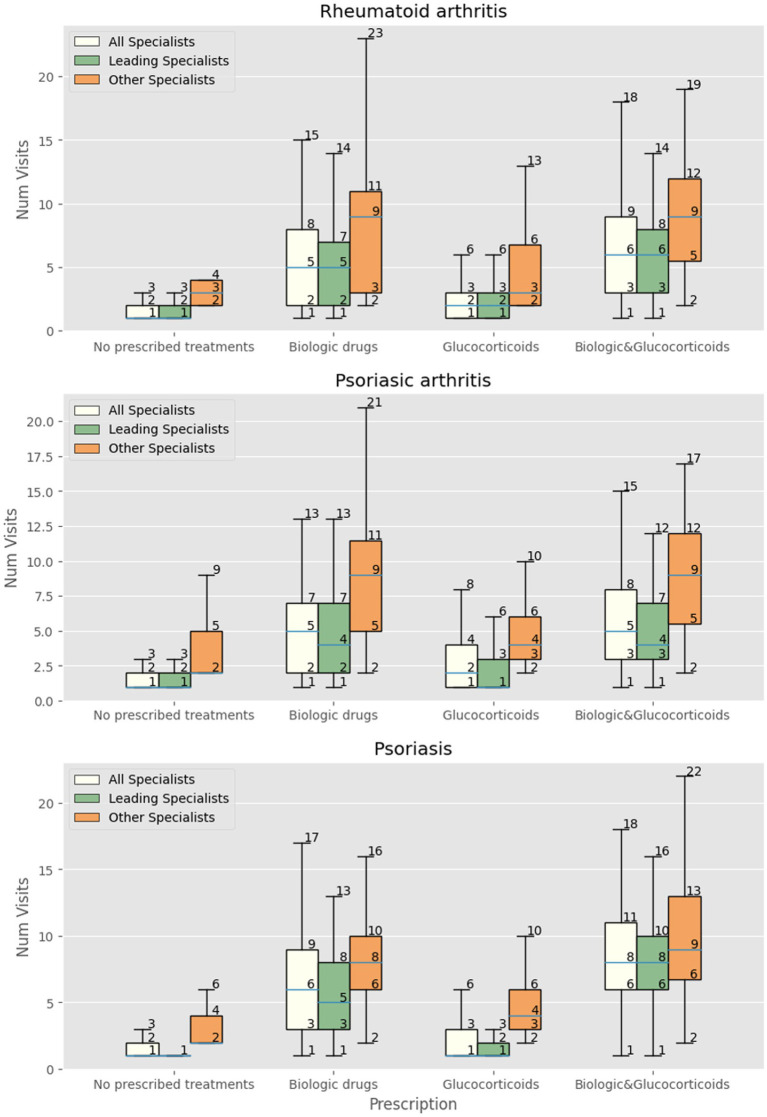
Number of visits with all specialists, leading specialist (dermatologist, rheumatologist, IBD-specialist, allergist), and other specialists in patients with RA, PsA and psoriasis receiving at least one prescription of biologics/small molecules, systemic and topic glucocorticoids, both biologics and glucocorticoids, or neither.

**Figure 2:**
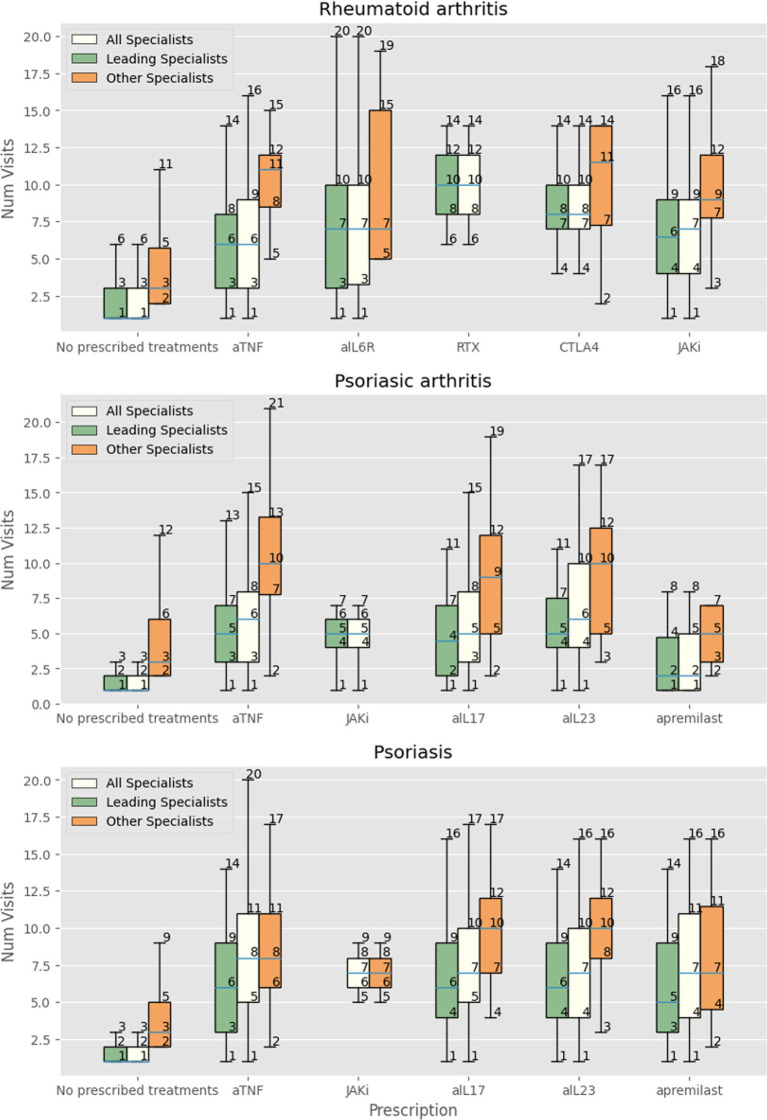
Number of visits with all specialists, leading specialist (dermatologist, rheumatologist, IBD-specialist, allergist), and other specialists in patients with RA receiving no treatment, at least one prescription of anti-TNF-alpha (aTNF), anti-IL-6 receptor (aIL6R), rituximab (RTX), abatacept (CTLA4), JAK inhibitors (JAKi), anti-IL-17 (aIL17), and IL-23 (aIL23), or apremilast.

We then focused the analysis on patients evaluated three or more times by specialists other than the leading one. Patients with RA were more likely to receive innovative drugs (OR 1.5, 95% CI 1.23–1.82) but not glucocorticoids (OR 0.99, 95% CI 0.89–1.1). The same was observed in patients with PsA, receiving more innovative drugs (OR 1.45, 95% CI 1.27–1.66) but not glucocorticoids (OR 1.04 95% CI 0.97–1.11). Patients with psoriasis received significantly more prescriptions for innovative drugs (OR 1.54 95% CI 1.4–1.7) and glucocorticoids (OR 1.07 95% CI 1.01–1.12). Figures 1, 2 show the relationship among the number of visits with any specialist, leading specialists, and other specialists, the use of glucocorticoids and innovative treatments, and the different mechanisms of action for innovative treatments, respectively.

The logistic regression analysis used to train the classifier to predict outcomes showed that the other specialists flag showed a positive correlation with the prescription of biological drugs, but if used in the classification with other features, it lost its original informativeness and may adjust the predictions of some observations by finding spurious correlations that are useful for the classification (*data not shown*). The data show that the classifications made with the number of visits have very similar performances to those obtained from all the available features while the improvement obtained by including the other specialists flag is very marginal (*data not shown*).

## Discussion

In our study natural language processing was applied to unstructured electronic clinical charts to analyze the impact of a multidisciplinary approach on treatment choices in RA, PsA and psoriasis cases in a large Center dedicated to immune-mediated diseases. Multidisciplinary care has been increasingly advocated in chronic inflammatory disorders, given the presence of comorbidities and associated conditions, and thus the growing need for more than one specialist contributing to the patient management. Comorbidities and associated conditions are well known for RA (24), but according to the latest recommendations the rheumatologist remains the primary specialist for patient care (25). In a similar manner, there is large consensus among experts on the need of multidisciplinary management for PsA, in particular with the collaboration of rheumatologists and dermatologists (26) to address all disease domains and ultimately reduce morbidity and mortality (24). The rising computational power of AI in complex disorders, including autoimmune rheumatic diseases, is expected to help clinicians in narrowing down the knowledge gaps found in complex cases, and to facilitate the incorporation of enormous data sets into clinical practice (27). Nonetheless, there is no clear evidence on the impact of this approach on patient management, particularly in terms of treatments, spanning from general options such as glucocorticoids to more innovative therapies such as biologics and targeted synthetic oral molecules. These two classes of medications are placed at the opposite sides of the treatment spectrum, thus reflecting a more complex clinical case series, as illustrated in difficult-to-treat RA (28) or other chronic conditions (29).

We report that patients with RA or PsA undergoing multiple evaluations are more likely to receive biologics or small molecules as well as glucocorticoids, with the former having the strongest correlation in patients visited more than three times by a rheumatologist. Such correlation is one of the crucial aspects of this study as it influences the choice of therapeutic approaches, and has already been demonstrated with the same natural language processing approach in the allergy setting at our ImmunoCenter (19). Further, patients evaluated by more than one specialist were also treated more frequently with biologics or small molecules, as well as glucocorticoids, thus possibly pointing at a more complex population requiring more innovative treatments. The associations we found (patients undergoing multiple evaluations and patients evaluated by more than one specialist were more likely to receive biologics or small molecules or glucocorticoids) may be in line with the recommendations of tight control and treat-to-target approach. In fact, multiple visits allow close of patient monitoring and prompt treatment when necessary, maximizing the likelihood of disease remission (30, 31). Further, the multidisciplinary approach indicates a comprehensive evaluation of the patient, with different aspects of the disease taken into account and treated by the dedicated specialist, as suggested by current recommendations (6, 9, 25). In our study, due to lack of data on clinical features and disease activity, this conclusion can only be speculative, as we may assume that patients seen more than once have a more severe disease, and require more frequent evaluations according to the treat-to-target approach proposed for RA and PsA (30, 31). The observed associations with glucocorticoids deserve a dedicated discussion, as these drugs can contribute to improve disease control but should be prescribed for limited periods in RA, with caution and at the lowest effective dose in PsA (5–7), while they have no indication in psoriasis (9).

It is difficult to compare our results with those of other real-word studies, as data analyzed are variable. However, studies have shown that visit frequency is associated with high disease activity, which also influences treatment intensification (32). Further, the number of conventional synthetic or biological disease-modifying antirheumatic drugs and glucocorticoids prescribed has been associated with a higher number of visits performed by a rheumatologist in patients with rheumatoid arthritis (33). Beyond the simple association between more visits and increased prescription of therapies, increased frequency of visits, a specific outpatient clinic, dedicated nurses, and patient information materials have been reported as useful tools for increasing adherence to treat-to-target guidelines (34), thereby improving patient outcomes.

Lack of data on the duration of therapies represents a first study limitation. Second, we do not know whether the increase in frequency of visits and prescriptions actually improved the morbidity and mortality in our cohort. Third, since no data on clinical features and disease activity have been included in our analysis, we cannot extimate the impact of multidisciplinary care on follow up.

Also, since the type of data used in this analysis are the clinical notes written after ambulatory hospital encouters, the amount of data that is possible to include in the analysis is limited to simple features of the patient journey. Specific data about the clinical state of the patient are not always reported and trying to include this kind of data would result in a sparsely filled dataset and in a non-reliable analysis. Not being able to gather more detailed data, including in particular clinimetrics and extraarticular manifestations or comorbidites is a limitation of this retrospective study. Analysis of similarities and differences in prescribing innovative treatments among the leading specialist and other specialists would require detailed data on clinical features, and could be the topic for prospective analyses. Despite these limitations, our data provide insights into the role of tight control and multidisciplinary approach in the management of patients with inflammatory arthropathies and psoriasis. The use of multiple integrated databases and sources of clinical information is expected to allow to perform further analysis and obtain more knowledge about the real-world impact of multidisciplinary care, thus helping clinicians to improve patient outcomes and advising for resource optimization. Further, current limits of available data such as the missing clinimetrics, may in the future be gathered from indirect proxies. We believe this is a crucial strength of our work: revealing the potential of innovative tools to improve the management of complex diseases with heterogeneous clinical manifestations and comorbidities. Science, technology, and clinical practice now produce large amounts of data and AI has unique capabilities to process and analyze the information, providing insights into diseases and their management. In our study we focused on clinical practice and multidisciplinary approach, but AI can integrate information from different sources. Elaboration of information from epidemiology, clinical manifestations, laboratory data on genomics, cytokines, immune cell profile, or histology can be relevant to identify approaches to prevent, early intercept, and treat rheumatic and dermatologic diseases. This would also enable patient stratification and personalized medicine, helping management and follow up. Further, it may suggest institution where to allocate resource for optimal management. Rheumatology and dermatology can benefit from this tool and their approaches can be completely revoluzionized in clinical and research practice (27, 35).

In conclusion, we demonstrated that a natural language processing and machine learning approach can be used to efficiently analyze high-dimensional data, reducing variability in classification and regression, confirming previously suspected associations, and most importantly recognizing patterns that are not usually detected by humans.

## Data availability statement

The datasets presented in this article are not readily available because of the regulations of our institution. Requests to access the datasets should be directed to dpo@humanitas.it and carlo.selmi@humanitas.it.

## Ethics statement

The study protocol was approved by the IRCCS Istituto Clinico Humanitas ethics committee and the patients gave their written informed consent for data analysis according to the mission of the IRCCS.

## Author contributions

FrM, FP, AC, MDS, AT, and CS collected the data. FrM, PM, MV, and FiM analysed the data. FrM, PM, MV, and CS wrote the manuscript. All authors contributed to the article and approved the submitted version.

## Funding

This work was partially supported by “Ricerca Corrente” funding from Italian Ministry of Health to IRCCS Humanitas Research Hospital.

## Conflict of interest

FP received reimbursements for lectures, presentations, speakers bureaus, and manuscript writing or educational events from AstraZeneca, Mundipharma, Menarini, Almirall, Chiesi, Valeas, Malesci Guidotti, Boehringer Ingelheim, Sanofi, GSK, Novartis, Stallergenes-Greer. CS received fees for consulting/speakers (AbbVie, Amgen, Alfa-Sigma, Biogen, Eli-Lilly, Galapagos, Janssen, Novartis, Pfizer, SOBI) and Research support (AbbVie, Amgen, Pfizer).

The remaining authors declare that the research was conducted in the absence of any commercial or financial relationships that could be construed as a potential conflict of interest.

## Publisher’s note

All claims expressed in this article are solely those of the authors and do not necessarily represent those of their affiliated organizations, or those of the publisher, the editors and the reviewers. Any product that may be evaluated in this article, or claim that may be made by its manufacturer, is not guaranteed or endorsed by the publisher.
