# Supplementary material for: Connecting the use of innovative treatments and glucocorticoids with the multidisciplinary evaluation through rule-based natural-language processing: a real-world study on patients with rheumatoid arthritis, psoriatic arthritis, and psoriasis
Source: Front Med (Lausanne). 2023 Jun 14;10:1179240. doi: 10.3389/fmed.2023.1179240 (PMC10301822; doi:10.3389/fmed.2023.1179240)
Supplement: Supplementary file 1 [file Table_1.docx]

**Supplementary Table 1**. Feature description for Glucocorticosteroids prescriptions across diseases taken into account. The rows regarding the ‘Gender’ feature express the number and percentage of female presence.

| **Disease** |  | **without GC** | **With GC** | **Overall** | **P-value** |
| --- | --- | --- | --- | --- | --- |
| **Rheumatoid arthritis** | **n** | 873 | 870 | 1743 |  |
|  | **Number of visits** | 1.0 [1.0 3.0] | 3.0 [1.0 6.0] | 2.0 [1.0 4.0] | <0.001 |
|  | **Gender** | 702 (80.4%) | 638 (73.3%) | 1340 (76.9%) | <0.001 |
|  | **Age** | 56.33 [46.5 68.37] | 60.585 [51.215 73.14] | 58.66 [48.83 71.29] | <0.001 |
| **Psoriatic arthritis** | **n** | 930 | 429 | 1359 |  |
|  | **Number of visits** | 1.0 [1.0 4.0] | 3.0 [1.0 6.0] | 2.0 [1.0 5.0] | <0.001 |
|  | **Gender** | 472 (50.8%) | 186 (43.4%) | 658 (48.4%) | 0.013 |
|  | **Age** | 53.95 [45.265 63.665] | 59.19 [49.23 69.37] | 55.52 [45.925 65.95] | <0.001 |
| **Psoriasis** | **n** | 1334 | 953 | 2287 |  |
|  | **Number of visits** | 1.0 [1.0 3.0] | 2.0 [1.0 6.0] | 2.0 [1.0 4.0] | <0.001 |
|  | **Gender** | 581 (43.6%) | 398 (41.8%) | 979 (42.8%) | 0.418 |
|  | **Age** | 50.0 [37.628 61.188] | 51.89 [38.91 62.96] | 50.62 [38.425 62.0] | 0.007 |

**Supplementary Table 2**. Feature description for small biologic drugs prescriptions across diseases taken into account. The rows regarding the ‘Gender’ feature express the number and percentage of female presence.

| **Disease** |  | **without bDMARDs** | **With bDMARDs** | **Overall** | **P-value** |
| --- | --- | --- | --- | --- | --- |
| **Rheumatoid arthritis** | **n** | 1286 | 457 | 1743 |  |
|  | **Number of visits** | 1.0 [1.0 3.0] | 6.0 [3.0 8.0] | 2.0 [1.0 4.0] | <0.001 |
|  | **Gender** | 990 (77.0%) | 350 (76.6%) | 1340 (76.9%) | 0.914 |
|  | **Age** | 59.45 [49.09 72.28] | 57.28 [47.6 67.76] | 58.66 [48.83 71.29] | <0.001 |
| **Psoriatic arthritis** | **n** | 875 | 484 | 1359 |  |
|  | **Number of visits** | 1.0 [1.0 2.0] | 5.0 [3.0 8.0] | 2.0 [1.0 5.0] | <0.001 |
|  | **Gender** | 426 (48.7%) | 232 (47.9%) | 658 (48.4%) | 0.835 |
|  | **Age** | 58.02 [48.39 68.585] | 51.86 [43.538 60.085] | 55.52 [45.925 65.95] | <0.001 |
| **Psoriasis** | **n** | 1630 | 657 | 2287 |  |
|  | **Number of visits** | 1.0 [1.0 2.0] | 7.0 [4.0 10.0] | 2.0 [1.0 4.0] | <0.001 |
|  | **Gender** | 721 (44.2%) | 258 (39.3%) | 979 (42.8%) | 0.034 |
|  | **Age** | 51.035 [38.702 62.528] | 49.5 [37.96 60.37] | 50.62 [38.425 62.0] | 0.051 |
